# How can the acceptance of artificial intelligence be transformed into employability? The sequential effect of a positive attitude toward artificial intelligence and career adaptability

**DOI:** 10.3389/fpsyg.2026.1808195

**Published:** 2026-04-17

**Authors:** Dan Shen, Min Xu, Xin Meng, Jie Sun, Zhifeng Zhou

**Affiliations:** 1 School of Environmental Science and Engineering, Yancheng Institute of Technology, Yancheng, China; 2 Department of Pediatrics, Yancheng Third People's Hospital, The Affiliated Hospital of Jiangsu Medical College, The Yancheng School of Clinical Medicine of Nanjing Medical University, Yancheng, China; 3 Mental Health Education Center, Yancheng Institute of Technology, Yancheng, China; 4 School of Mathematics and Physics, Yancheng Institute of Technology, Yancheng, China; 5 School of Materials Science and Engineering, Yancheng Institute of Technology, Yancheng, China

**Keywords:** generative artificial intelligence acceptance, positive attitude towards artificial intelligence, career adaptability, students' perceived employability, PLS-SEM

## Abstract

As artificial intelligence is increasingly embedded into education and career development contexts and its applications grow in higher education, how student acceptance of artificial intelligence affects student perception of employability is a major research topic. Based on SCCT, we see generative AI acceptance (GAIA) as a situational occupational resource and study how it affects students' perceived employability (SPE) through positive AI attitude (PAAI) and career adaptability (CA). We used PLS-SEM to model 689 Chinese students. We found that acceptance of artificial intelligence was positively associated with positive attitudes toward artificial intelligence, which in turn promote career adaptability, and subsequently with students' perceived employability. Acceptance of artificial intelligence also indirectly affects perceived employability through successive positive attitudes toward artificial intelligence and career adaptability. This paper further strengthens the theoretical impact of SCCT on digital technology. The findings indicate that acceptance of artificial intelligence was positively associated with perceived employability, both directly and indirectly through positive attitudes toward artificial intelligence and career adaptability. Practically, it emphasizes that universities must develop vocational adaptability resources of students, beyond just technological applications, to support students' readiness to work in AI-driven jobs.

## 1 Introduction

The transition from higher education to labor market is an important step for global graduates (Tomlinson, 2024). The transition from higher education to labor market has become increasingly complex and uncertain because of the rapid advancement of artificial intelligence and digitalization. Countries around the world are working together to address these challenges. One way is to integrate artificial intelligence into higher education to improve student employability (Martin et al., 2025). The widespread use of generative artificial intelligence has changed organizational production modes, and also affected employment structures and career trajectory in short term (Hui et al., 2024). Research indicates artificial intelligence is changing job market and impacting individuals perceptions of career development and employment cognition (Tschang and Almirall, 2021; Kong et al., 2023; Bonfiglioli et al., 2025). In modern higher education, generative AI tools are increasingly integrated into career planning and employability Development courses. Students use AI to explore career options, refine resumes, and analyze labor market trends. But there is still a question: How does AI acceptance affects perceived employability, as it affects their perception of employability? Few studies have explored the impact of AI acceptance on career outcomes (Maulana et al., 2025) and career planning (Martin et al., 2025) and how AI acceptance affects college students' perceived employability is unclear.

To understand the relation between artificial intelligence acceptance and students' perceived employability, we developed research based on SCCT theory. According to SCCT, career outcomes evolve through situational support, cognitive evaluation, self-regulating resources and career outcomes (Lent et al., 1994; Lent and Brown, 2013). In this paper, acceptance of AI is considered contextual technical support, positive attitudes toward AI are considered cognitive evaluation, career adaptability is seen as self-regulating resources and perceived employability as occupational outcomes. We argue that accepting artificial intelligence promotes vocational adaptability resources by improving positive attitudes, ultimately increasing students' perceived employability.

**Figure 1:**
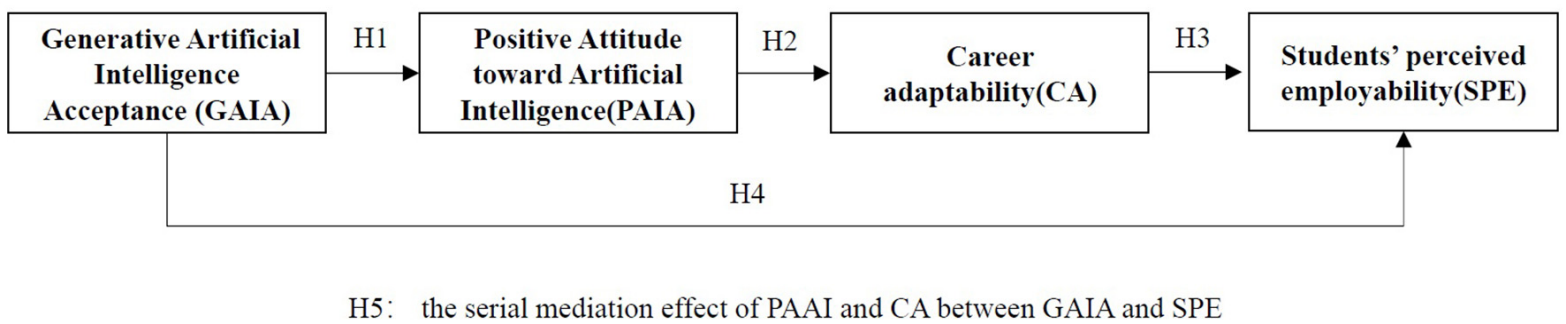
Conceptual research framework.

This paper seeks to clarify our understanding of the interaction between generative artificial intelligence acceptance, positive attitude toward artificial intelligence, career adaptability and students' perceived employability (Figure 1). It contributes in three main ways. First, acceptance of artificial intelligence is integrated into the study of the antecedents of employability. It broadens the technical context of existing employability research. While previous studies have explored the antecedents of employability (Ho et al., 2023), few studies have investigated artificial intelligence. While some studies consider the relation of career adaptability and employability (Koen et al., 2012), there is little study linking artificial intelligence acceptance with career adaptability. Second, social cognitive career theory is expanded to be understood in the digital technology context. In the past studies, we have not explored the possibility of antecedents of SCCT theory for identifying perceived employability. Third, we focus on how artificial intelligence affects career cognition through psychological resources. Fourth, we provide a theoretical foundation and policy implications to universities on how to guide students whose perceptions of employability and adaptive resources are still developing in terms of how artificial intelligence can improve their readiness for the labor market.

## 2 Literature review and hypotheses development

### 2.1 Social cognitive career theory

Social Cognitive Career Theory (SCCT) is derived from Social Cognitive Theory (SCT) and describes how individuals develop careers, make career choices and achieve career development goals in their careers (Lent et al., 1994). According to SCCT, careers are not dictated by static personality traits or external environmental factors. Career development is characterized by cognitive factors such as self-efficacy beliefs, outcome expectations and career goals, learning experiences, situational support or obstacles. Combining cognitive and social factors, SCCT offers a framework for understanding how individuals form careers, make career choices and achieve career goals (Lent et al., 1994).

Career development is dynamic self-regulating process based on situational support, cognitive evaluation, adaptive resources. Situational factors help an individual adapt to vocational tasks by shaping cognitive evaluations such as self-efficacy beliefs and outcome expectations leading to career outcomes. Unlike theories based on behavioral intention or single behavioral outcome, SCCT emphasizes professional ability, adaptive resources and subjective career development outcomes which explain very clearly the complex dynamic and uncertain career and employment landscape of today.

SCCT has been used to study college student employability, vocational adaptability, vocational self-efficacy, and psychological adaptation in new vocational settings (Lent et al., 2008; Lent and Brown, 2013). SCCT has become increasingly important theoretical framework for career development research. Based on this, we use SCCT to study artificial intelligence situational factors to influence undergraduate student perceived employability through cognitive evaluation and vocational adaptation resources.

### 2.2 Generative artificial intelligence acceptance (GAIA) and positive attitude toward artificial intelligence (PAAI)

Technology acceptance typically refers to an individual's acknowledgment and ongoing adoption of a particular technology. It serves as a crucial prerequisite for the successful dissemination and sustained use of technology (Davis, 1989; Taherdoost, 2018). In the realm of generative artificial intelligence GAIA denotes the extent to which individuals recognize and integrate AI tools within educational or organizational settings. This acceptance is expressed through a comprehensive assessment of the technology's accessibility, usability, and functional value in real-world scenarios.

PAAI reflects a favorable view of AI's utility, legitimacy, and future development potential (Schepman and Rodway, 2022). Generative AI liberates individuals from routine activities and repetitive tasks. Enhanced acceptance and trust, coupled with improved knowledge, education, and analytical skills (Schiavo et al., 2024), can positively influence people's attitudes toward AI.

There have been studies on the relation between technology acceptance and attitude. Results show that an individual's performance expectations, effort expectations and situational support conditions influence his/her decision to adopt technology (Davis, 1989; Venkatesh et al., 2003). In artificial intelligence, a person's attitude toward AI is also strongly related to its experience with the technology. Schepman and Rodway (2022) emphasized trust in AI influence overall evaluations. Kaya et al. (2022) and Schiavo et al. (2024) found that individuals with higher education and more Internet usage experience generally have more positive attitude toward AI. This suggests that frequency and level of technological exposure may be important to influence attitudes toward AI.

This paper examines GAIA as a precursor to PAAI. Previous work shows that individual's attitudes toward technology and value judgement can influence technology adoption and use (Davis, 1989; Ismatullaev and Kim, 2022; Kelly et al., 2023). In schools where generative AI is well integrated, a student's mastery experience during continuous use can lead to positive expectations about the technology's utility (Morosan, 2011), which leads to stable positive attitudes toward AI. According to SCCT, situational support shapes attitudes by influencing outcome expectations and cognitive processing. Therefore, we expect more acceptance of AI will lead to positive evaluations of technology.

This study proposes the hypothesis:

H1: GAIA is positively associated with PAAI.

### 2.3 Positive attitude toward artificial intelligence (PAAI) and career adaptability (CA)

Career adaptability refers to the psychological resources individuals use to deal with change in their career and uncertainty. It includes four dimensions: career concern, career control, career exploration and career confidence (Savickas, 2005, 2013). Career concern refers to planning future careers, career control refers to assuming career responsibility and shape one's future. Career exploration refers to actively seeking new roles and opportunities, and career confidence refers to belief in achieving career goals and solving problems (Savickas and Porfeli, 2012). Studies show higher levels of career adaptability help individuals deal with occupational changes and uncertainty better (Zacher et al., 2015).

A number of cognitive and psychological factors can influence career adaptability. Self-efficacy, future orientation and emotional regulation are related to career adaptability (Rudolph et al., 2017; Zacher et al., 2015). This suggests career adaptability develops through cognitive processing of situations and behavioral investment and not alone.

In this paper we consider the cognitive variable PAAI, which represents the positive attitude of an individual regarding the utility, rationality and future potential of artificial intelligence (Schepman and Rodway, 2020, 2022). PAAI represents value judgments and emotions that individuals make in technological context. In career development, a positive attitude toward artificial intelligence can encourage students to get involved in technology related learning and career exploration, helping them to be prepared for changing technologies.

From the perspective of SCCT, cognitive evaluation is the link between situational support and ability development. Attitude is the cognitive variable that influences the ability formation of an individual by changing their outcome expectations and behavior (Lent et al., 1994; Lent and Brown, 2013). Persons who have positive attitudes toward artificial intelligence are more likely to consider technological changes as opportunities for growth rather than threats, which helps them to plan their career, control technology, and explore and grow careers through participation. Thus, when one sees AI as a development opportunity rather than an external threat, students are more likely to commit to learning and career exploration.

Based on this, this study proposes the following hypotheses:

H2: PAAI is positively associated with CA.

### 2.4 Career adaptability (CA) and students' perceived employability (SPE)

Perceived employability is generally defined as an individual's subjective assessment of the ability to find, retain and transform jobs on the labor market (Clarke, 2018; Tomlinson, 2012). Unlike objective skills or actual employment outcomes, perceived employability is an individual's cognitive assessment of their own competitiveness and market value, as a cognitive variable of occupational outcomes.

Career adaptability is a psychological tool that combines planning, control, exploration and confidence in career changes and uncertainty (Savickas, 2005, 2013). Research indicates that vocational adaptability is crucial for transitioning from academia to the workforce (Konstam et al., 2015; Van der Horst et al., 2017) and strongly influences employment, career success and satisfaction (Rudolph et al., 2017; Johnston, 2016). Furthermore, persons with high vocational adaptability tend to actively seek employment, thus increasing their chances of employment success (Koen et al., 2012). Together these studies show that career adaptability not only helps transitioning from academia, but also increases labor readiness by promoting proactive occupational behavior.

According to SCCT (Lent et al., 1994; Lent and Brown, 2013, 2019), career development results from the interaction between situation support, cognitive variables and self-regulation. Higher self-efficacy beliefs and outcome expectations can lead to higher career adaptability, which reduces the perceived threat of environmental uncertainty and makes individuals more likely to view the work process as planned, manageable and achievable goal. Therefore, career adaptability affects behavioral input and directly determines subjective assessment of employability. In generative AI, higher vocational adaptability students plan their career paths, take career decision-making duties, explore new opportunities, and feel confident in their ability to tackle complex tasks (Duong et al., 2024). The accumulation of these psychological resources further strengthens their positive assessment of employment competitiveness and market adaptability (Grosemans and De Cuyper, 2021).

Based on this, this study proposes the following hypotheses:

H3: CA is positively associated with SPE.

### 2.5 Generative artificial intelligence acceptance (GAIA) and students' perceived employability (SPE)

In addition to sequentially promoting PAAI and CA, generative artificial intelligence acceptance may also directly influence students' perceived employability. According to SCCT, individuals' beliefs about their capacity to engage with contextual resources contribute not only to adaptive behavior but also to self-evaluative career judgments (Lent et al., 1994). In technology-driven environments, perceived readiness to adopt and use emerging tools may be a prominent psychological resource shaping individuals' employability perceptions (Lent and Brown, 2013).

Students who view generative AI as accessible, useful and manageable may interpret acceptance as evidence of technological competence and future capability (Ho and Le, 2026). Since AI-related competencies are increasingly integrated into the current labor markets, acceptance of AI may directly influence students' self-assesses of competitiveness and career readiness (Pham Thi, 2025; Zhang et al., 2025), independent of attitude or adaptability pathways. Beyond its potential influence through PAAI and career adaptability, GAIA may also directly influence SPE.

H4: GAIA is positively associated with SPE.

### 2.6 The role of positive AI attitude (PAAI) and career adaptability (CA) in mediating the relationship between AI acceptance (GAIA) and students' perceived employability (SPE)

Existing research indicates that artificial intelligence can influence employment structures and career opportunities (Bonfiglioli et al., 2025; Gao et al., 2025). Moreover, studies show that AI can enhance students' learning engagement and career awareness (Zhang et al., 2025), providing a positive psychological environment for career development (Kong et al., 2023). However, how AI acceptance impacts students' perception of employability through ongoing cognitive and skill development is not explored.

According to SCCT, GAIA is considered to be a contextual technical support. PAAI is an individual's cognitive evaluation and emotional attitude toward the technological context. CA is the self-regulation resource developed by the individual during career development. SPE is the proximally career outcome cognition (Lent et al., 1994; Lent and Brown, 2013).

The acceptance of AI in professional learning can shape the values and attitudes of students toward professionals. Students who can easily integrate AI technology in career planning and employment guidance can be more likely to engage with AI, and thus have positive outcome expectations and emotional attitudes (Bandura, 1986). High acceptance of AI by students can increase participation in related activities, boost positive assessment of AI, promote vocational adaptability resources and ultimately improve SPE.

Second, artificial intelligence positive attitude encourages students to engage actively in technological environment for career learning and job exploration. By repeating participation and experience accumulation, individuals gradually improve self-efficacy and readiness to act (Wang et al., 2023; Buniel et al., 2025). Existing studies show occupational flexibility increases readiness to act and ability to deal with problems in complex situations (Rudolph et al., 2017).

Third, professional adaptability is also important link to employability formation. Professional adaptability increases the individual's ability to take action and tackle challenges in challenging situations, thus increasing their subjective judgment of employment availability and competitiveness (Hirschi, 2012; Rudolph et al., 2017). Research shows that employability formation depends on individual adaptability and proactive resources (Fugate et al., 2004; Clarke, 2018). Artificial intelligence enhances students' confidence in their employment readiness (Xiao and Zheng, 2025; Maulana et al., 2025). Artificial intelligence not only highlights the convenience of professional activities but also boosts students' enthusiasm for such activities.

Based on the above inferences, this study proposes:

H5: PAAI and CA sequentially mediate the relationship between GAIA and SPE.

## 3 Research methodology and results

### 3.1 Sample and procedure

The study was conducted during the Spring 2025 semester at a single comprehensive university in China. Data were collected from undergraduate students enrolled in an elective career planning course involving career exploration, labor market analysis, resume writing, and the introduction of generative artificial intelligence tools such as ChatGPT for career learning tasks. The use of AI tools was optional and did not constitute a formal intervention. Only undergraduate students who voluntarily agreed to participate were included in the study.

Questionnaires were distributed during scheduled class sessions. Participation was voluntary and confidential, and the research was approved by the Ethics Committee of the university. Data were encoded before analysis in order to remove personal information. Out of 702 students who completed the questionnaire, 689 were retained and analyzed, after removing incomplete responses, yielding an effective response rate of 98.1%. Age ranged from 18 to 22 years, average age 18.68 and standard deviation of 1.018.

In order to avoid common method bias, we addressed several procedural issues. Respondents were told that there were no right answers and that they would only use their responses for research purposes and not for evaluation purposes. The survey was completed independently, which reducing social desirability bias and evaluation fear.

### 3.2 Measures

In this study, all measurements were based on previous relevant research and evaluated using a five-point Likert scale, ranging from one (strongly disagree) to five (strongly agree).

The acceptance of generative AI (with Cronbach's alpha 0.959) was measured using 15 items adopted by Yilmaz et al. (2023), containing four dimensions: Performance Expectations, Effort Expectations, Facilitators, and Social Impact. Examples include statements like: “I think generative AI applications are very useful in daily life” and “Generative AI applications are easy to use”.

The positive attitude toward artificial intelligence (Cronbach's alpha = 0.765) is adapted from four affirmative items by Schepman and Rodway (2020) to assess students' favorable views on artificial intelligence. An example project includes “Artificial Intelligence is Exciting.”

Career adaptability, with a Cronbach's alpha of 0.953, was evaluated using a 12-item short version of the Career Adaptability Scale. This scale is derived from the original 24-item version developed by Savickas and Porfeli (2012). It assesses four dimensions: concern, control, curiosity, and confidence, with each dimension comprising three items (Magalhães et al., 2015). An example item is

“I have a good understanding of the requirements for my future career.”

Perceived employability, with a Cronbach's alpha of 0.940, was assessed using six items adapted from Pitan and Muller (2019). An example statement is:

“I am confident that my personal qualities will make it easy for me to secure a graduate-level job.”

The control variables consist of age, gender, part-time work experience, and region. Age is categorized into groups: 1 for 18 years old, 2 for 19 years old, 3 for 20 years old, 4 for 21 years old, and 5 for 22 years old. Gender is coded as 1 for male and 2 for female. Part-time work experience is indicated by one for no and two for yes. Regional classification is coded as 1 for urban and 2 for rural.

As shown in Table 1, the proportion of women is higher than that of men. Most of the students have part-time work experience, and more than half of the students come from rural areas.

**Table 1:** The demographic participants.

S/No.	Characteristic	Frequency	Percentage
1	**Gender**	689	
	Female	434	63.0
	Male	255	37.0
2	**Age**	689	
	18	430	62.4
	19	116	16.8
	20	86	12.5
	21	49	7.1
	22	8	1.2
3	**Part-time work experience**	689	
	No	83	12.0
	Yes	606	88.0
4	**Region**	689	
	City	295	42.8
	Rural	394	57.2

All survey instruments were written in Chinese. For scales written in English, standard translation and back-translation procedures were used to check language and concepts alignment. First, items were translated into Chinese by bilingual researcher, and back-translated into English by bilingual expert. Any differences were discussed and resolved. A pilot test was performed with 30 undergraduate students before survey, test item clarity and comprehensibility, and make minor wording adjustments to make it more appropriate for Chinese students. Pilot subjects were not included in the final sample.

### 3.3 Methodology used

To test the relationship between research hypotheses and structural model proposed here, partial least squares structural equation modeling (PLS-SEM) is used for modeling. PLS-SEM is suitable for prediction-oriented research and complex mediation models, which estimate direct effects and indirect effects simultaneously (Hair et al., 2019), since we consider sequential mediation and want to maximize explained variance of endogenous constructs, PLS-SEM was a suitable model estimation method.

PLS-SEM can model data that might differ from multivariate normality and asymmetric distributions (Becker et al., 2022). Preliminary results indicated minor deviations from normality, supporting PLS-SEM which does not require strict distribution assumptions. Collinearity diagnostics were assessed using variance inflation factors (VIF) (Kock, 2015), to detect multicollinearity issues. All VIF values were below the recommended level and no collinearity concerns were raised. PLS-SEM can handle path models and can provide stable estimation results in mediation (Memon et al., 2021).

Although GAIA was initially developed as a multidimensional construct, the present study modeled it as a single reflective construct because the dimensions demonstrated strong internal consistency and conceptual unity. Accordingly, the indicators were treated as reflective manifestations of a common latent variable.

All constructs were modeled as reflective measurement models because the indicators represent underlying latent variables consistent with the literature. The PLS algorithm was implemented in SmartPLS (version 4.0) using the path weighting scheme. The maximum number of iterations was set to 300, with a stop criterion of 1*e*^−7^ to ensure convergence stability. For the analysis of structural paths and mediating effects, we bootstrapped 5,000 samples and constructed bias-corrected confidence intervals, as Hair et al. (2019) recommends. In addition to *R*^2^ and effect sizes (*f*^2^), global fit was evaluated using standard root mean square residual (SRMR) (Henseler et al., 2016).

### 3.4 Descriptive statistics

To verify the measurement model, the reliability and validity of all potential variables in the model were evaluated. Table 2 reports the means, standard deviations and correlation coefficients of the main variables. The relevant analysis results show that there is a significant correlation among the acceptance of artificial intelligence, the positive attitude toward artificial intelligence, career adaptability and perceived employability, providing a preliminary empirical basis for the subsequent structural model test.

**Table 2:** Descriptive statistics and correlations.

Construct	Mean	SD	1	2	3	4	5	6	7	8
1 Gender	1.370	0.483	**1**							
2 Age	1.680	1.018	−0.029	**1**						
3 Work experience	1.880	0.326	0.117^**^	−0.240^**^	**1**					
4 Region	1.570	0.495	−0.096^*^	0.107^**^	−0.005	**1**				
5 GAIA	3.954	0.567	−0.013	0.090^*^	−0.01	−0.038	**1**			
6 PAAI	3.782	0.532	−0.079^*^	0.039	0.062	−0.061	0.611^**^	**1**		
7 CA	4.003	0.496	0.031	0.016	0.062	−0.054	0.531^**^	0.577^**^	**1**	
8 SPE	3.785	0.650	−0.001	0.093^*^	−0.057	−0.032	0.465^**^	0.448^**^	0.666^**^	**1**

^*^p < 0.05.

^**^p < 0.01.

GAIA, Generative artificial intelligence acceptance; PAAI, Positive attitude toward artificial intelligence; CA, Career adaptability, SPE, Students' perceived employability. Bold values indicate the correlation coefficients.

### 3.5 Measurement model

This measurement model is evaluated from four dimensions: internal consistency reliability, convergent validity, discriminant validity and multicollinearity. Tables 3–5 report the relevant values.

**Table 3:** Convergent validity.

Constructs	Items	Content	Factor loadings	VIF	Cronbach's alpha	CR	AVE
GAIA					0.959	0.960	0.637
Performance expectancy	PE1	I find generative AI applications useful in my daily life.	0.723	3.922		
	PE2	The use of generative AI applications increase my chances of achieving the things that are important to me.	0.767	3.889		
	PE3	Generative AI applications help me get things done faster.	0.764	4.569		
	PE4	Using generative AI applications increase my productivity.	0.770	2.643		
Effort expectancy	EE1	Learning how to use generative AI applications is easy for me.	0.787	2.706		
	EE2	Generative AI applications are easy to use.	0.796	3.059		
	EE3	My interaction with generative AI applications is clear and understandable.	0.829	3.252		
Facilitating conditions	FC1	Generative AI applications are compatible with other technologies I use.	0.829	3.371		
	FC2	I can get help from others when I have difficulties in using generative AI applications.	0.822	3.077		
	FC3	If I experience any problems while using generative AI applications, I can access the necessary information for a solution.	0.843	3.535		
Social influence	SI1	People important to me think I should use generative AI applications.	0.836	3.352		
	SI2	The people I model my behavior on think I should use generative AI applications.	0.803	3.808		
	SI3	People whose opinions I value prefer me to use generative AI applications.	0.818	4.574		
	SI4	People who are important to me are using generative AI applications.	0.790	3.449		
	SI5	People who are important to me encourage the use of generative AI applications.	0.782	3.791		
PAAI					0.765	0.774	0.584
	PAAI1	I am interested in using artificially intelligent systems in my daily life	0.779	1.393			
	PAAI2	Artificial Intelligence is exciting	0.727	1.458			
	PAAI3	There are many beneficial applications of Artificial Intelligence	0.780	1.520			
	PAAI4	I would like to use Artificial Intelligence in my own job	0.770	1.521			
**CA**					0.953	0.953	0.659
Concern	CONC1	Thinking about what my future will be like	0.786	3.117			
	CONC2	Realizing that today's choices shape my future	0.761	3.127			
	CONC3	Preparing for the future	0.818	3.511			
control	CONT1	Making decisions by myself	0.783	2.385			
	CONT2	Taking responsibility for my actions	0.833	3.952			
	CONT3	Doing what's right for me	0.853	4.442			
Curiosity	CURI1	Looking for opportunities to grow as a person	0.867	3.744			
	CURI2	Investigating options before making a choice	0.803	2.565			
	CURI3	Becoming curious about new opportunities	0.824	3.014			
Confidence	CONF1	Performing tasks efficiently	0.750	2.007			
	CONF2	Overcoming obstacles	0.822	3.715			
	CONF3	Solving problems	0.836	3.928			
SPE					0.940	0.944	0.768
	SPE1	I am confident that through my personal qualities, it will be easy for me to get a graduate-level job	0.838	2.657			
	SPE2	I feel confident that I will be able to find appropriate work after leaving the university	0.874	3.248			
	SPE3	I feel confident about making applications to organizations of interest	0.887	3.125			
	SPE4	I am aware of the employment opportunities open to me	0.889	3.557			
	SPE5	I have good knowledge about the requirements of my future career	0.886	3.542			
	SPE6	I am generally confident of success in job interviews	0.885	3.378			

CR, composite reliability; AVE, average value extracted; GAIA, generative artificial intelligence acceptance; PAAI, positive attitude toward artificial intelligence; CA, career adaptability; SPE, students' perceived employability.

**Table 4:** Discriminant validity-Fornell–Larcker criterion.

Construct	CA	GAIA	PAAI	SPE
CA	0.812			
GAIA	0.527	0.798		
PAAI	0.591	0.628	0.764	
SPE	0.674	0.470	0.454	0.877

Bold values on the diagonal represent the square root of AVE for each construct. GAIA, generative artificial intelligence acceptance; PAAI, positive attitude toward artificial intelligence; CA, career adaptability, SPE, students' perceived employability.

**Table 5:** Discriminant validity-heterotrait-monotrait.

Construct	CA	GAIA	PAAI	SPE
CA				
GAIA	0.555			
PAAI	0.683	0.719		
SPE	0.705	0.490	0.531	

GAIA, generative artificial intelligence acceptance; PAAI, positive attitude toward artificial intelligence; CA, career adaptability, SPE, students' perceived employability.

Internal consistency reliability was evaluated using Cronbach's alpha coefficient and composite reliability (CR). All constructs demonstrated strong reliability (Fornell and Larcker, 1981): the Cronbach α coefficients all exceeded the recommended threshold of 0.7 (ranging from 0.765 to 0.959), and the CR values all exceeded the recommended threshold of 0.70 (ranging from 0.774 to 0.960).

Aggregated validity was evaluated using the mean extracted variance (AVE). The AVE value range is from 0.584 to 0.768, all exceeding the recommended minimum value of 0.50, indicating satisfactory aggregation validity.

Discriminant validity was evaluated using the Fornell–Larcker criterion and the heterogeneity—unity ratio (HTMT). The Fornell–Larcker results show that the square root of AVE of each construct is greater than its correlation coefficient with other constructs. Meanwhile, the HTMT values were all below the conservative threshold of 0.85 (ranging from 0.490 to 0.719), indicating sufficient discriminative validity between constructs (Fornell and Larcker, 1981; Hair et al., 2019).

To evaluate the potential common method bias, this paper adopts the total collinearity test proposed by Kock (2015) to calculate the total collinearity variance inflation factors of each construct. The results show that the VIF of all constructs is below the conservative threshold of five, indicating that the common method bias is unlikely to have a substantial impact on the research conclusion.

Overall, this measurement model demonstrates satisfactory reliability and validity and can be used for subsequent structural model verification.

### 3.6 Structural model analysis

The structural model is evaluated by testing collinearity, explanatory power (*R*^2^), effect size (*f*^2^), predictive correlation (*Q*^2^), and the significance of the hypothetical relationship.

**Figure 2:**
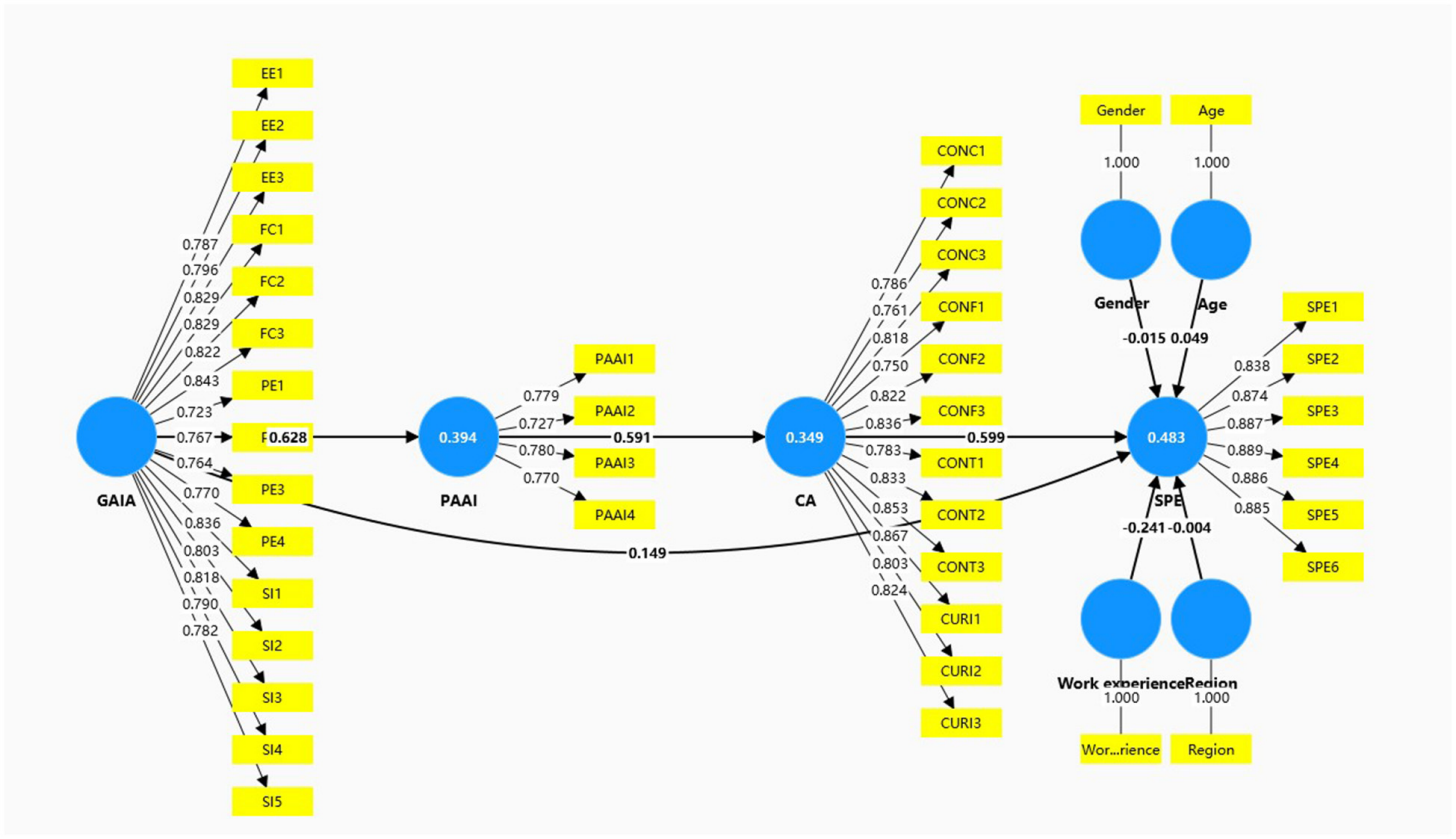
PLS-SEM results.

The coefficient of determination (*R*^2^) results indicate that the model has a moderate level of explanatory power for endogenous variables. Specifically, the structural model explains 39.4% of PAAI variation, 34.9% of the CA variation, and 48.3% of the SPE variation. According to the judgment criteria of Hair et al. (2019), the explanatory level of SPE of the model in this study can be regarded as medium, and the explanatory level of CA is between weak and medium. Overall, the model in this study demonstrates satisfactory explanatory and predictive capabilities in predicting students' perceived employability. In addition, the global model fit was assessed using the standardized root mean square residual (SRMR). The SRMR value was 0.079, which is below the recommended threshold of 0.08, indicating a good model fit. Figure 2 presents the structural model results, including the path coefficients and indicator loadings.

Effect size analysis (*f*^2^) also shows the relative contributions of each predictor variable to the endogenous variable. CA has large effect size (*f*^2^ = 0.497) on SPE, suggesting that it plays an important role in explaining students' perception of employability. GAIA effect size (*f*^2^ = 0.650) on PAAI indicates that AI acceptance plays a key role in shaping positive attitudes toward artificial intelligence. PAAI also has large effect size (*f*^2^ = 0.536) on CA. According to the judgement criteria proposed by Hair et al. (2019), the key paths in this study all have strong explanatory contributions. In contrast, the direct effect of GAIA on SPE demonstrates a small effect size (*f*^2^ = 0.031). The control variables (gender, age, region, and work experience) show negligible effect sizes on SPE.

The predictive correlation is evaluated by the *Q*^2^ value. The results show that the *Q*^2^ values of each endogenous variable are all greater than zero, among which the *Q*^2^ values of CA, PAAI, SPE are 0.228, 0.222, and 0.333, respectively. According to the judgment criteria proposed by Hair et al. (2019), the model in this study demonstrated a moderate level of predictive correlation on CA and PAAI, and approached a relatively high predictive level on SPE. This indicates that the model not only has a robust performance at the interpretation level, but also shows good potential for external sample prediction at the prediction level, which conforms to the methodological requirements of PLS-SEM prediction-oriented analysis.

In addition, Harman's single-factor test was conducted to assess potential common method variance. The first unrotated factor accounted for 47.21% of the total variance, which is below the commonly suggested 50% threshold, indicating that common method bias is unlikely to be a serious concern.

### 3.7 Hypothesis testing

The structural model is evaluated by the path coefficient (β) and the *p*-value of the hypothesized relationship in the study. Table 6 reports these values.

**Table 6:** Results of hypothesis testing.

Hypotheses	Path	Path coefficients (β)	*t*-Statistics value	*P*	Hypothesis Result	95% CI	*f*^2^ value
H1	GAIA → PAAI	0.628	17.794	< 0.001	Supported	[0.557, 0.697]	0.650
H2	PAAI → CA	0.591	17.286	< 0.001	Supported	[0.526, 0.656]	0.536
H3	CA -> SPE	0.599	14.919	< 0.001	Supported	[0.518, 0.675]	0.497
H4	GAIA → SPE (direct effect)	0.149	3.707	< 0.001	Supported	[0.072, 0.230]	0.031
H5 (indirect effect)	GAIA → PAAI → CA → SPE						
	0.222	8.696	< 0.001	Supported	[0.176, 0.275]		

GAIA, generative artificial intelligence acceptance; PAAI, positive attitude toward artificial intelligence; CA, career adaptability, SPE, Students' perceived employability.

As the hypothesis suggests, GAIA has a significant positively associated with students' PAAI (β = 0.628, *t* = 17.794, *p* < 0.001), supporting hypothesis H1. Furthermore, PAAI was positively correlated with CA (β= 0.591, *t* = 17.286, *p* < 0.001), which verified H2. Consistent with previous career development studies, CA has a significant positively associated with SPE (β= 0.599, *t* = 14.919, *p* < 0.001), verifying H3.

Regarding the mediating effect, the Bootstrapping results indicated that GAIA had a significant indirect impact on SPE through the sequence path of PAAI and CA [β= 0.222, *t* = 8.696, *p* < 0.001, 95% CI (0.176, 0.275)]. Moreover, the confidence interval does not contain zero, which strongly supports the H5 hypothesis.

In addition to sequential indirect pathways, GAIA direct association with SPE was estimated to determine mediation structure. GAIA strongly correlates with SPE (β= 0.149, *t* = 3.707, *p* < 0.001). Further sequential indirect effects of GAIA on SPE through PAAI and CA remain significant. Both direct and indirect effects are statistically significant, and hence support partial mediation structure rather than full mediation.

Including the direct GAIA → SPE path increased the explained variance of SPE from 45.4 to 47.3% (Δ*R*^2^ = 0.019), indicating incremental explanatory contribution beyond the mediated pathway.

### 3.8 Control variables and robustness analysis

To assess model stability, gender, age, work experience, and region were used as controls of SPE. They showed that they had relatively small effects on SPE and did not change the magnitude, direction or significance of the main structural relationships. After adding control variables, the explained variance of SPE increased slightly from 47.3 to 48.3% (Δ*R*^2^ = 0.010) suggesting that the mechanism remains stable. Work experience had a negative impact on SPE (β= −0.241, *p* < 0.001), gender and region were not significant predictors. Age had small positive effect. All these factors did not alter the hypothesis for the model.

Multi-Group analysis (MGA) was performed across gender groups. Such structural paths did not differ significantly between male and female students (all *p* > 0.05) indicating overall model invariance across gender. The effect of PAAI was significantly stronger among male students (β_male_ = 0.664; β_female_ = 0.444; *p* < 0.001). This suggests that the translation of favorable AI attitudes into adaptive career resources may be more easily translated among male students.

## 4 Discussion

This paper considers the psychological transformation of generative artificial intelligence for students' careers. Based on SCCT, the results show that PAAI as a context enabling factor boosts SPE by encouraging PAAI and building CA resources.

GAIA was positively associated with PAAI as well (Cengiz and Peker, 2025; Kaya et al., 2022; Schiavo et al., 2024). GAIA includes performance expectations for AI, effort expectations, facilitating conditions and social effects (Yilmaz et al., 2023). AI acceptance, in SCCT, is an effective setting (Bandura, 1986), encouraging positive learning experiences and reducing cognitive barriers, which then shapes individuals' opinions on technologies. Students who repeat AI practices become more positive, and see AI-related activities as manageable and achievable, and can see AI-related activities as attainable. Also, when AI values and practices are taken as personal cognition, the normative support and environmental empowerment they provide helps students to perform professional and employment tasks effectively. GAIA was linked to more positive attitudes toward AI, which may reflect differences in self-belief and outcome expectations.

Further analysis shows that PAAI was significantly associated with CA with respect to previous studies linking AI-related attitudes to career development (Burhan, 2024; Kong et al., 2023). This supports the notion that PAAI as a psychological factor influences career-related behavior and ability development (Presbitero and Teng-Calleja, 2022). In SCCT, PAAI is a cognitive-emotional tool that governs how individuals interpret situations and learning experiences. When students recognize the practicality, controllability, and affordability of artificial intelligence, sense of situational disorder diminishes, increasing self-belief, and positive career achievement expectations. These cognitions correspond closely with the four dimensions of CA: relevance (present future direction of career development), sense of control (present initiative in career decision making), curiosity (present active exploration of career options), and self-confidence (posite belief in solving career problems). By strengthening these adaptive resources, PAAI empowers students to deal with career challenges, thus establishing a psychological foundation for improving their perception of employability.

This paper finds CA demonstrated the strongest statistical association with SPE, and thus, it can play a key role in shaping employability in AI environments, and this is consistent with previous results (Koen et al., 2012). CA, unlike GAIA and PAAI, directly represents the person's ability to manage change, handle uncertainty, and shape career development in a changing labor market (Zacher et al., 2015). Thus, CA has more explanation power for SPE, since it includes concerns about the future, control over career decisions, tendency to explore new opportunities and confidence in problem solving. Technical experience alone is not a guarantee of employment advantage. CA is psychological hub that enables individuals to translate positive AI experiences into confidence and control over their employment prospects (Savickas and Porfeli, 2012).

This work confirms that PAAI and CA mediate the relationship between GAIA and SPE. This suggests the “context-cognitive-adaptation-outcome” mechanism promoted by SCCT and broadens the explanation of employability formation for artificial intelligence. Artificial intelligence practices were associated with differences in students' attitude and vocational adaptability gradually by creating an environment conducive to innovation and exploration. When artificial intelligence is a routine part of learning or organization, normative support and situational empowerment of artificial intelligence help students to feel more comfortable and more easily perceive professional behavior as a manageable process. Therefore, mediation pathways of PAAI and CA illustrate how artificial intelligence indirectly promotes SPE formation through normalization and empowerment.

In addition to the sequential mediation mechanism, GAIA strongly relates directly to SPE, suggesting that AI acceptance may also act as a psychological resource. From a social cognitive career theory perspective, seeing AI as accessible and manageable may stimulate students' confidence in entering technology-driven labor markets. This implies that GAIA contributes to employability perceptions beyond attitudinal and adaptability mechanisms.

Given the cross-sectional design of this study, findings should be interpreted as consistent with theoretical associations rather than definitive causal relationships. Although the proposed sequential pathways are grounded in social cognitive career theory, existing data are insufficient to establish temporal order or causality. Future research employing longitudinal studies, experimental designs, or cross-lagged analyses is needed to validate the proposed directional framework.

## 5 Conclusion and implications

### 5.1 Conclusion

This study examined the psychological associations linking GAIA and SPE. The findings support a serial mediation model in which GAIA is linked to PAAI, which in turn strengthens CA, corresponding with higher SPE. Rather than relying primarily on a direct technological influence, AI acceptance appears to shape perceived employability largely through an attitudinal and adaptive pathway.

Students who accept AI have more positive cognitive–affective attitudes toward it, which lead to career adaptive resources like concern, control, curiosity, and confidence which help students navigate uncertainty in a rapidly changing labor market. AI acceptance is an analogy resource that translates into employability perceptions through adaptive self-regulatory powers.

Practically, the results suggest that higher education institutions should not limit AI education to technical training alone, instead foster a psychologically supportive and empowerment AI learning environment for students' adaptability resources. By embedding AI practices into career development curricula, universities can transform technological exposure into employability gains.

### 5.2 Theoretical contributions

This paper, grounded in social cognitive career theory, investigates how acceptance of generative artificial intelligence affects students'perceived employability through cognitive and adaptive resources, giving new theoretical perspectives on employability development in artificial intelligence.

First, acceptance of the generative artificial intelligence is integrated into perceived employability and extends technical context aspects of employability research. Previous studies have examined the relationship between vocational adaptability and employability (Koen et al., 2012) and the effect of vocational development learning on employability (Ho et al., 2023). However, there are no systematic tests to measure whether acceptance of artificial intelligence can influence employability through psychological resources. This paper addresses this gap. Second, it strengthens the explanatory power of SCCT in digital technology context by supporting dynamic interaction process emphasized by SCCT. Accepting AI can facilitate psychological transformation through sequential development of positive AI attitudes and career adaptability resources. Thirdly, it offers contextual evidence in Chinese universities. With the rapid introduction of artificial intelligence technology in higher education, this paper provides valuable empirical support to show how digital technology together with attitudes and adaptive resources affect students' employability.

### 5.3 Practical implications

The results can be useful for colleges and universities to improve students' employability in artificial intelligence. Students accept artificial intelligence and feel it affects their employability, positive attitude and vocational adaptability. Colleges and universities should provide technical tools and skills training, but also provide a supportive, standardized and empowering environment for AI use. By integrating artificial intelligence into teaching, learning tasks and career development guidance, colleges can help students experience technology and improve confidence in career tasks.

Second, the results show that career adaptation is required to bridge artificial intelligence and employability. Colleges and universities should combine technical training with career development education when designing courses and projects. For example, by focusing on context tasks, projects, careers simulations, they should teach students to view artificial intelligence as a resource to explore career paths, manage uncertainties, plan for future and not just a tool for efficiency. This adaptive resource-based training can help students translate technical knowledge into employability skills.

From the educational management and policy perspective, we found that decision makers favor artificial intelligence but also consider its effects on student psychological resources and career awareness. By encouraging artificial intelligence applications that encourage innovation and tolerate trial and error, universities can help students develop positive attitudes toward technology and professional adaptation, leading to easy transition into labor market.

### 5.4 Research limitations and future research directions

While we have done great work with the theory and empirical data presented in this paper, there are some limitations to be addressed. First, the cross-sectional design of this paper limits our ability to determine causality in the dynamic evolution of acceptance of AI, positive attitudes toward AI, career adaptability, and perceived employability. Future work should consider longitudinal designs or cross-lag models to study how AI experiences affect student perception of career adaptation resources and employability over time. In addition, because all variables were collected through self-report measures at a single time point, common method variance cannot be entirely ruled out. Future research may adopt longitudinal or multi-source designs to further mitigate potential method bias. Second, the sample is a sample of students from Chinese universities. While this provides practical information, the results could be applied in different countries, educational systems or labor market settings. Future work could further test the validity of these conclusions in different countries through cross-cultural comparisons or multi-national samples. Third, while we construct a mechanism of action based on SCCT, we do not measure some key theoretical aspects (specific types of learning experiences, intensity of situational support or objective employment outcomes). Future work could include more specific situational variables or combine objective employment indicators like employment quality and job matching degree to better understand how different AI applications effect career development together. Finally, Future work could explore how different AI applications impact career development together to better understand how technological features and individual psychological resources jointly shape employability.

## Data Availability

The original contributions presented in the study are included in the article/supplementary material, further inquiries can be directed to the corresponding author.
